# A head-to-head comparison of SCRalyze and Ledalab, two model-based methods for skin conductance analysis

**DOI:** 10.1016/j.biopsycho.2014.08.006

**Published:** 2014-12

**Authors:** Dominik R. Bach

**Affiliations:** a Department of Psychiatry, Psychotherapy, and Psychosomatics, University of Zurich, Switzerland; b Wellcome Trust Centre for Neuroimaging, University College London, United Kingdom

**Keywords:** SCR, EDA, GSR, Model-based method, Biophysical model, Model inversion

## Abstract

Model-based analysis of skin conductance responses (SCR) can furnish less noisy estimates of sympathetic arousal (SA) than operational peak scoring approaches, as shown in previous work. Here, I compare two model-based methods for analysis of evoked (stimulus-locked) SCR, implemented in two software packages, SCRalyze and Ledalab, with respect to their sensitivity in recovering SA. Four datasets are analysed to compare predictive validity, i.e. the sensitivity to distinguish pairs of SA states that are known to be different. SCRalyze was significantly better able than Ledalab to recover this known difference in four out of five tested contrasts and comparable in the remaining one. SCRalyze performed significantly better than conventional analysis in all contrasts. I conclude that the model-based method engendered in SCRalyze is currently the best available approach to provide robust and sensitive estimates of sympathetic arousal.

## Highlights

• Comparison of two developed methods for model-based analysis of phasic skin conductance responses.
• Four datasets are used to compare predictive validity.
• SCRalyze shows higher predictive validity than Ledalab in 4 of 5 comparisons, and equal predictive validity in the fifth.
• SCRalyze shows higher predictive validity than peak-scoring in all comparisons.

## 1 Introduction

Skin conductance responses (SCR) are commonly used to index a central state of sympathetic arousal (SA) (Bach, 2014, Boucsein, 2012). During the past two decades, model-based analysis of SCR has seen a surge of interest (Bach & Friston, 2013). Initially, the necessity to separate SCR peaks in fast event-related paradigms (Barry, Feldmann, Gordon, Cocker, & Rennie, 1993) fostered the mathematical formalisation of physiological models which describe how sudomotor nerve (SN) activity causes SCR (Alexander et al., 2005, Lim et al., 1997). This allows estimation of SN activity as an index of SA, with potentially better time resolution than SCR (Benedek and Kaernbach, 2010a, Benedek and Kaernbach, 2010b). A further step was the development of mathematical neural models describing how SA causes SN activity. This allows the direct estimation of SA from SCR (Bach et al., 2010a, Bach et al., 2011, Bach et al., 2009). One possible benefit of this model-based approach is a possibility to reduce the impact of noise on indices of sympathetic arousal (SA), which can enhance statistical sensitivity. I have previously analysed theoretically under which conditions a method might be able to suppress the impact of measurement and physiological noise, and of psychological processes unrelated to an experiment (i.e. latent noise) (Bach & Friston, 2013). I have also demonstrated empirically that all model-based methods engendered in the software SCRalyze, developed by myself with colleagues, are more sensitive than conventional peak-scoring approaches (Bach et al., 2010a, Bach et al., 2011, Bach et al., 2013). The present paper provides a head-to-head comparison of two publicly available model-based methods with respect to their sensitivity in inferring evoked (stimulus-locked) SA from observed SCR: the general linear convolution modelling (GLM) method implemented in the software SCRalyze (Bach et al., 2009, Bach et al., 2013), and several approaches implemented in the software Ledalab (Benedek and Kaernbach, 2010a, Benedek and Kaernbach, 2010b). These methods have been introduced and discussed in detail elsewhere. In the present paper, I briefly summarise the major differences between these methods and conventional analysis, and then empirically compare their sensitivity.

### 1.1 Conventional analysis

The aim of conventional analysis is to find “indices” of SCR data that closely follow the central SA state of interest. For example, to index stimulus-evoked SA, one will usually filter the data to discard observation noise, define a response window after the stimulus, and define some criteria do detect peaks within this window. The goal of this process is to ensure that SA not evoked by the stimulus is largely kept out of the analysis (e.g. it falls outside the defined response window) and that data features that do not correspond to stimulus-evoked SA are also largely discarded (e.g. these data features do not fulfil peak criteria). For details of how to best achieve this goal, the reader is referred to the current recommendations by the Society for Psychophysiological Research (Boucsein et al., 2012).

### 1.2 Model-based analysis

Model-based approaches use explicit, mathematical models that formulate psychophysiological assumptions on how observed data are generated by central processes. For example, one can posit a model that formulates the relation SA → SCR. This kind of model, in general, is often termed a “forward model”: it is a physiological model that predicts data time series (SCR) from a known central process (SA). However, in analysis of experimental data we are faced with the opposite situation: we know the observed SCR data but not SA, and seek to estimate the SA time series that generated these SCR data. In order to do so, one has to turn this forward model backwards, to arrive at the relation SA ← SCR. In statistics, this process is often termed “model inversion”, and it provides estimates of SA, given SCR. Both conventional and model-based analysis seek to infer SA from SCR. The difference is that model-based methods use a more stringent mathematical language and computational methods to do so, while the general aim is the same.

### 1.3 Peripheral model

All available model-based methods split up the relation SA → SCR into two relations: SA → SN → SCR. The peripheral model SN → SCR is a biophysical model that specifies how SN activity generates SCR, usually in the form SCR = SN⊗SCRF, where ⊗ is the convolution operator, and SCRF is a “canonical” skin conductance response function. This model is, in a basic version, deterministic. This means that SCR time series, in this model, is only influenced by SN and not by other factors. This is of course a coarse approximation to reality. SCR data contains noise, and deterministic deconvolution enhances noise (Benedek & Kaernbach, 2010b). Different model inversions schemes treat this problem differently. Formally, the peripheral forward models of SCRalyze and Ledalab are very similar. The SCRalyze model and its parameters were derived phenomenologically (i.e. by curve-fitting to a large number of data sets). The Ledalab model is based on an explicit biophysical model, and its parameters are optimised for each individual data set.

### 1.4 Ledalab

Ledalab uses two approaches to invert the peripheral model, termed by the authors “Discrete Deconvolution Analysis” (DDA) (Benedek & Kaernbach, 2010b) and “Continuous Deconvolution Analysis” (CDA) (Benedek & Kaernbach, 2010a). The reader is referred to the original papers for an explanation of these names, and for technical details. In a nutshell, CDA performs a deterministic inversion of the peripheral model, and thus arrives at an estimated SN time series. This SN time series will contain noise, and could even contain negative numbers, which have no biophysical interpretation (nerve firing cannot be “negative”). To render SN time series better interpretable, DDA was developed which ensures that SN time series contain no meaningless negative values: it decomposes the SCR data into a positive-valued SN time series and a remainder time series. The latter is assumed by the authors to reflect the pore-opening process (Benedek & Kaernbach, 2010b) according to the pore-valve model (Edelberg, 1993). Having arrived at an estimated SN time series by either CDA or DDA, one now needs to estimate SA. To do so, Ledalab uses “indexing”, just as in conventional analysis. The estimated SN time series is filtered, and some criteria are used to identify peaks in defined response windows. For technical details, the reader is referred to the original papers, and to the methods section.

### 1.5 SCRalyze

SCRalyze uses a similar peripheral model as Ledalab (Bach, Flandin, Friston, & Dolan, 2010). However, this model is not inverted on its own. Instead, SCRalyze also defines a forward model SA → SN, which defines how SA generates SN activity. For example, stimulus-locked arousal is assumed to elicit an instantaneous burst of SN firing at a constant latency after the stimulus (Bach et al., 2009), and the model does not contain any other SN firing. This means that the model SA → SN → SCR is not deterministic any more, because much of the SCR time series cannot be explained by stimulus-locked SA. Hence, SCRalyze performs probabilistic inversion – a standard approach in statistics. This procedure estimates the most likely SA parameters, given the model and observed data. In this statistical framework, the unexplained features of the data are regarded as observation error. For technical details, the reader is referred to the original paper and the methods section. Note that in this framework there is no need for “indexing” SA, because SA is directly estimated from the data.

### 1.6 Data conditioning

Skin conductance data contain tonic drifts not caused by phasic SN activity. In order to avoid assigning such signals to phasic SA, different approaches are used. SCRalyze band pass filters the signal explicitly. Ledalab, on the other hand, removes the tonic signal in an iterative procedure aimed at model-based decomposition of tonic and phasic components: initial deterministic inversion of the peripheral model (both for CDA and DDA) yields an SN time series from which phasic activity is detected and removed. The remaining “tonic” SN time series is re-convolved with the peripheral response function and the result subtracted from the data.

### 1.7 Methods comparison

One may find theoretical arguments for or against particular methods – the interested reader is referred to a previous review (Bach & Friston, 2013). In the context of experimental data analysis, however, the crucial question is which method is best able to recover the (unknown) SA – I have termed this “predictive validity”. The analysis of predictive validity rests on assumptions about the SA that is truly elicited by experimental events. This is of course a psychological construct and not directly measurable. In this paper, I follow the approach to experimentally create conditions which are known to invoke categorically different states of SA, for example high and low SA (Bach et al., 2013). One can then ask how well a method recovers this categorical difference by establishing the evidence for a model in which SA estimates for two states are drawn from distributions with different mean rather than the same mean (i.e. as in a *t*-test). Comparison of non-nested models in the statistical literature is done by comparing estimates of model evidence. Here, I use the Log Bayes Factor (LBF) as a measure of relative model evidence, where a smaller LBF indicates better model evidence, and in the present context this means higher predictive validity. An absolute LBF difference of >3 is often regarded as decisive, because it corresponds to a classical *p*-value of 0.05: if a classical test statistic falls into the rejection region, the probability of the null hypothesis being true is *p* = 0.05 and of the alternative hypothesis being true is *p* = 0.95. Hence, the null hypothesis is 19 times less likely than the alternative hypothesis; ln(19) ≈ 3 (Penny et al., 2004, Raftery, 1995).

Given the fundamental differences between SCRalyze and Ledalab, it is not known which approach yields the more robust estimates of phasic SA. Here, I compare them in their predictive validity. I analyse data from four experiments according to the currently recommended settings for both methods packages.

## 2 Methods

### 2.1 Datasets

I re-analyse four datasets, all acquired during a study that examined the influence of distracting background noise on the perception of emotionally arousing images. There was no interaction of acoustic distractors with differential SCR in any experiment. The main effects of auditory stimulation will be reported elsewhere. For the present analysis, all data were collapsed across the auditory stimulation factor. Two of the four datasets were also used in a previous technical note (Bach et al., 2013). Participants were recruited from student and general population via an online recruitment system. Local Research Ethics Committees approved all experiments.

The first experiment investigated SCR in response to negative-arousing and neutral IAPS pictures. 60 healthy individuals (30 male, 30 female; age: *M* = 23.7; SD = 4.7 years) participated in this experiment. They watched, in randomised order, the 45 least arousing neutral (valence within 1 standard deviation around the mean) and 45 most arousing aversive pictures (valence lower than 1 standard deviation below the mean) from the International Affective Picture Set [IAPS] (Lang et al., 2005) for 1 s each, with an inter stimulus (ISI) interval randomly sampled from 7.65 s, 9 s, or 10.35 s. Each picture was presented once. Participants were instructed to press the cursor up or down key on a computer keyboard to indicate whether they liked the picture or not. The experiment was divided into three blocks with 45 s breaks in between.

The second experiment was similar to the first, with an additional third condition of positive arousing images. 40 healthy individuals (20 male, 20 female; age: *M* = 21.9; SD = 3.8 years) watched the 16 least arousing neutral, most arousing aversive and most arousing positive images (defined analogous to experiment 1, and excluding explicit nude images) from the IAPS, for 1 s each, in randomised order, and in one single block. ISI was 4.4 s. Responses were the same as in experiment 1. Two datasets were excluded: one due to technical malfunction, one due to lack of compliance with the instruction to rate the images by key press.

The third experiment examined SCR to emotional face expression. 42 healthy individuals (21 male, 21 female; age: *M* = 25.2; SD = 4.0 years) watched photographs from 38 actors of the Karolinska Directed Emotional Faces set (Lundqvist, Flykt, & Öhman, 1998) with angry, fearful, and neutral expression. The faces were masked to remove hair and clothing, and shown in grey scale on a black background. The experiment was divided into three blocks. Event timing and responses were the same as in experiment 1.

Experiment 4 included all neutral pictures from experiment 1 with the same event timing and responses, and was divided into 3 blocks. 61 healthy individuals (31 male, 30 female; age: *M* = 25.7; SD = 4.5 years) took part. Lists of images, used in these experiments, are available from the author.

### 2.2 SCR recordings and pre-processing

I recorded skin conductance on thenar/hypothenar surface of the non-dominant hand using 8 mm Ag/AgCl cup electrodes and 0.5%-NaCl electrode paste (GEL101; Biopac Systems). In experiments 1 and 2, I used a custom-build constant voltage coupler (2.5 V). The output of the coupler was converted into an optical pulse frequency with an offset (i.e. minimum sampling rate) of 100 Hz. The pulse signal was digitally converted and recorded (Micro1401/Spike 2, Cambridge Electronic Design). In experiment 3, I used an integrated SCR coupler/amplifier/AD converter (GSR100 C/MP150/AcqKnowledge 4, Biopac). An integrated SCR coupler/amplifier (LabLinc V71-23, Coulbourn) and AD converter (DI-149/Windaq, Dataq) were used in experiment 4.

SCR data were filtered with a unidirectional 1st order Butterworth low pass filter with a cut off frequency of 5 Hz. Data were then down sampled to 10 Hz. The data were visually checked for artefacts, but no formal artefact rejection was implemented.

### 2.3 Estimation of SA

Analysis of stimulus-locked responses was done using the current recommendations for both software packages.

*SCRalyze* (scralyze.sourceforge.net): I used the general linear convolution model (GLM) approach as recommended for stimulus-locked (evoked) responses. Each event type in the experiment was modelled as a Dirac delta function centred on the event onset, convolved with a canonical skin conductance response function (SCRF) and its first derivative (Bach, Flandin, et al., 2010), to construct a design matrix (Bach et al., 2009). Data and design matrix were filtered with a unidirectional 1st order Butterworth high pass filter with cut off frequency of 0.05 Hz, according to current recommendations (Bach et al., 2013). The resulting data time series was *z*-transformed for each participant. From the estimated amplitude parameters for the canonical SCRF and its derivative, the response for each condition was reconstructed (Bach et al., 2013) by multiplying both the canonical response, and its derivative, with the respective parameter estimate and adding them. The peak of highest absolute amplitude was identified, and its signed amplitude extracted as an estimate of mean SA in this experimental condition. This SA estimate can be regarded as the sum of a true SA value, which is always non-negative, and some estimation error; the error term can be negative. Hence, the SA estimate can take any value, including zero or negative values.

*Ledalab* (www.ledalab.de): This method package does not recommend one single estimate of SA but offers a choice of 4 measures that were all analysed, without correction for multiple comparison. I applied both discrete decomposition analysis (DDA) (Benedek & Kaernbach, 2010b) and continuous decomposition analysis (CDA) (Benedek & Kaernbach, 2010a). I extracted SN peaks within a response window of 1–4 s after stimulus onset, and used an amplitude threshold of 0.01 μS for response scoring, following the approach of the two validation papers. Two initial values were considered in the optimisation, corresponding to the default setting in Ledalab. The respective SA indices for each method are: sum of SCR amplitudes of above-threshold SCRs (DDA 1 and CDA 1, termed “AmpSum” in the software package), sum of SCR area of above-threshold SCRs (DDA 2, “AreaSum”), and average phasic driver (CDA 2, “SCR”). These measures were then averaged across trials including zero responses, within each condition, as estimates of mean SA in this experimental condition.

*Secondary Ledalab analysis*: To explore the impact of removing between-subject variance, two supplemental Ledalab analyses were performed. One analysis was done on *z*-transformed individual SCR data. In the other analysis, individual trial-by-trial SA indices from Ledalab were *z*-transformed across all trials from one subject, before averaging within conditions.

*Peak scoring*: For comparison, I computed an operational index of SA as recommended by the Society for Psychophysiological Research (Boucsein et al., 2012), with a post-stimulus response window of 1–3 s (Barry, 1990, Boucsein, 2012, Dawson and Filion, 2007), and a post-onset peak window of 0.5–5 s (Boucsein, 2012). Onset SCR values were subtracted from peak SCR values, and SCR magnitude was computed as SA index by averaging all responses including zero responses. I have previously shown that this operational index has higher predictive validity than SCR amplitude (excluding zero responses) or magnitude based on a response window of 1–4 s (Bach et al., 2013); hence this is the best available peak scoring index among those currently recommended.

### 2.4 Development of the hypothesis

Analysis of predictive validity rests on assumptions on ground truth, i.e. the SA that is truly elicited by stimuli. These assumptions about ground truth can for example be derived from the extant literature using operational analysis. This is not the same as assuming operational measures to reflect ground truth (i.e. assessing concurrent validity). Instead, operational measures are assumed to be noisy as well, but informative about categories of states with different SA.

There is strong evidence in the operational literature that negatively and positively arousing pictures elicit stronger SA than neutral pictures (Boucsein, 2012). Hence, I analysed the contrasts aversive > neutral pictures (experiments 1 and 2) and positive > neutral pictures (experiment 2). SCR to emotional face expression have been reported rather inconsistently in the extant literature (Banks et al., 2012, Clark et al., 1992, Conty et al., 2010, Springer et al., 2007, Vrana and Gross, 2004); in particular there is no consistent evidence that negative facial expression elicits stronger SA than neutral expression. I therefore used an independent peak scoring approach to define a contrast of interest. This analysis revealed stronger responses to fearful than to angry faces (*T*(41) = 2.65; *p* = 0.01). This categorical difference was then assumed to reflect ground truth, and I analysed how well the two model-based methods could capture this difference. Finally, dataset 4 was used to investigate the ability of these methods to separate stimulus-locked responses from a condition without stimulation, under the assumption that neutral pictures elicit phasic orienting responses. To this end, I created dummy events, which were then used for all analyses. These dummy events were inserted between each two trials and their onsets drawn from a normal distribution with a mean of 4.5 s after the previous stimulus onset, and a standard deviation of 0.75 s. Dummy events were analysed as a separate experimental condition, termed “no picture”.

I hypothesised that the two methods under study would differ in their sensitivity to recover these known SA differences between two conditions in different contrasts.

### 2.5 Statistical analysis

Sensitivity of SCR analysis methods to recover a known ground truth has been cast as model comparison (Bach et al., 2010a, Bach et al., 2013), as classification problem (Bach, Friston, et al., 2010), or as search for the highest test statistics for a given contrast (Bach et al., 2009, Barry, 1990). These approaches are all equivalent in determining the most sensitive method, but the model comparison approach also allows a principled statement of whether a method is significantly more or less sensitive than another method. Hence, I report sensitivity in terms of a log Bayes factor (LBF) – the difference in negative log model evidence between a given model and a reference model. For simplicity (because SCRalyze reports just one SA estimate, and Ledalab reports 4), SCRalyze was used as reference model. According to the definitions used here, lower LBF indicates higher model evidence, i.e. higher sensitivity to distinguish the two experimental conditions. An absolute LBF difference larger than 3 is often considered decisive as it corresponds to a *p*-value of 0.05.

Specifically, I used a general linear model with the contrast of interest as the response variable, and the estimated SA as predictor. The design matrix included subject effects. This is equivalent to a paired *t*-test and thus tests whether SA estimates for the two different states are drawn from distributions with different means. This approach allows computing a residual sum of squares RSS, which was converted to a negative log likelihood value LL, such that smaller LL values indicate a higher predictive validity using the following relation taken from (Burnham & Anderson, 2004)

(1) $$\text{LL}=n\log\left(\frac{1}{n}\text{RSS}\right)$$

where *n* is the number of observations. This disregards model complexity, which was the same for all analyses. LBF is the difference in LL between a given method and the reference method.

I also computed paired *t*-tests for each contrast and method, to facilitate an intuitive understanding of the difference between the methods. LBF and *t*-value are monotonically related – higher *t*-values translate to lower LBF and indicate higher sensitivity.

### 2.6 Specificity

Finally, while sensitivity is generally desirable for any method of inferring SA, one reviewer pointed out that this may come at the cost of reduced specificity. Specificity in the present context refers to the ability to reject a difference between two states of SA if there is none. Specificity might be reduced either by the fact that noise is different between two conditions and spuriously assigned to different SA – a possibility that is difficult to test without hypotheses on the type of noise that could cause this problem. Another cause of reduced specificity is if a method systematically yields differences between experimental conditions, e.g. based on the order in which they are entered into the model. There is no theoretical reason to expect this for any of the methods. I empirically confirmed this by randomly assigning trials from the aversive condition from experiment 1 into two groups, and testing for differences between them. This was done, for each method, 1000 times. As both reflect samples from a distribution with the same mean, one would expect a significant finding at the alpha rate, i.e. at 5%, approximately 50 significant findings for each method.

A question not addressed in this paper is whether a method is able to detect SA in a single experimental condition. I have previously argued that such analysis can easily be biased: if a method adds a constant term to SA estimates, it would appear to better detect the known SA. This could for example happen by methods that exclude negative SA values, such as peak scoring and some Ledalab methods (but not the method termed CDA 2 in this paper). This is why the present work analyses sensitivity only in terms of differences between experimental conditions.

## 3 Results

**Fig. 1:**
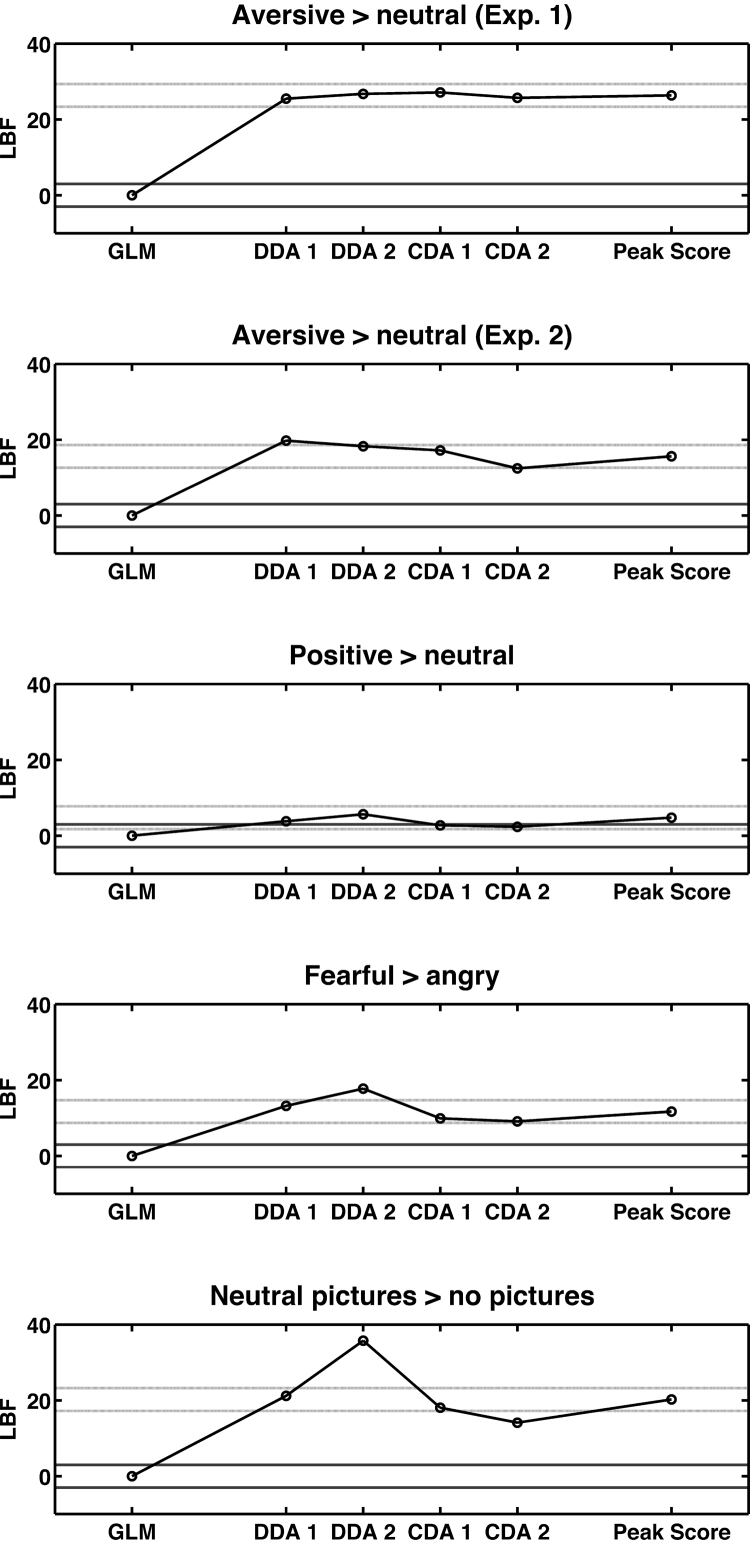
Predictive validity, expressed as Log Bayes Factors (LBFs), for the comparison of two experimental conditions. The two conditions are assumed to differ in sympathetic arousal (SA) such that lower LBF-values indicate better sensitivity of the respective method to recover that difference. An absolute LBF difference of more than 3 is usually considered significant and corresponds to a classical *p*-value of *p* < .05. LBFs are expressed with reference to the SCRalyze GLM. Dark grey lines indicate an absolute LBF of 3. Dotted light grey lines indicate an absolute LBF difference of 3 from the peak scoring approach, allowing comparison of Ledalab measures with peak scoring. GLM: estimated SA amplitude from the general linear model in SCRalyze. DDA 1: sum of estimated SCR amplitudes of significant SCRs from non-negative deconvolution in Ledalab; DDA 2: sum of estimated SCR area of significant SCRs from non-negative deconvolution in Ledalab, CDA 1: sum of estimated SCR amplitudes of above-threshold SCRs from continuous deconvolution in Ledalab; CDA 2: average estimated phasic driver from continuous deconvolution in Ledalab.

LBF values are shown in Fig. 1, and *t*-values in Table 1. In four out of five contrasts, SA estimates from the GLM implemented in SCRalyze were significantly more sensitive than any Ledalab measure, often with a wide margin. In the fifth contrast, CDA measures were not significantly different from SCRalyze, while DDA was again significantly less sensitive.

**Table 1:** Comparison of predictive validity. The table shows Log Bayes Factors (LBFs), for the comparison of two experimental conditions. The two conditions are assumed to differ in sympathetic arousal (SA) such that lower LBF-values indicate better sensitivity of the respective method to recover that difference. LBFs are expressed with reference to the SCRalyze GLM. An absolute LBF difference of more than 3 is usually considered significant and corresponds to a classical *p*-value of *p* < .05. *t*-Scores for paired *t*-tests on the condition differences are given in brackets. GLM: estimated SA amplitude from the general linear model in SCRalyze. DDA 1: sum of estimated SCR amplitudes of significant SCRs from non-negative deconvolution in Ledalab; DDA 2: sum of estimated SCR area of significant SCRs from non-negative deconvolution in Ledalab, CDA 1: sum of estimated SCR amplitudes of above-threshold SCRs from continuous deconvolution in Ledalab; CDA 2: average estimated phasic driver from continuous deconvolution in Ledalab.

		Aversive > neutral (Dataset 1)	Aversive > neutral (Dataset 2)	Positive > neutral (Dataset 2)	Fearful > angry (Dataset 3)	Neutral picture > no picture (Dataset 4)
		LBF (*t*_59_)	LBF (*t*_37_)	LBF (*t*_37_)	LBF (*t*_41_)	LBF (*t*_60_)
*SCRalyze*	GLM	0.0 (7.8)	0.0 (6.7)	0.0 (3.1)	0.0 (4.7)	0.0 (8.5)

*Ledalab*						
Initial analysis	DDA 1 (Amp Sum)	25.5 (4.4)	19.8 (3.4)	3.8 (2.2)	13.2 (2.3)	21.2 (5.7)
	DDA 2 (Area Sum)	26.8 (4.2)	18.3 (3.6)	5.7 (1.7)	17.8 (0.8)	35.8 (3.6)
	CDA 1 (Amp Sum)	27.1 (4.1)	17.2 (3.8)	2.7 (2.5)	9.9 (3.0)	18.1 (6.2)
	CDA 2 (SCR)	25.7 (4.4)	12.5 (4.6)	2.4 (2.6)	9.1 (3.2)	14.1 (6.7)

*z*-Transformed data	DDA 1 (Amp Sum)	23.4 (4.7)	17.8 (3.7)	4.6 (2.0)	15.3 (1.8)	20.8 (5.8)
	DDA 2 (Area Sum)	30.1 (3.7)	18.8 (3.5)	8.4 (0.4)	15.9 (1.6)	27.4 (4.9)
	CDA 1 (Amp Sum)	25.2 (4.4)	12.2 (4.7)	3.0 (2.4)	5.3 (3.9)	19.9 (5.9)
	CDA 2 (SCR)	22.9 (4.8)	12.0 (4.7)	3.7 (2.2)	1.9 (4.4)	16.3 (6.4)

*z*-Transformed results	DDA 1 (Amp Sum)	8.5 (6.7)	15.5 (4.1)	4.9 (1.9)	5.9 (3.8)	18.9 (6.0)
	DDA 1 (Area Sum)	11.9 (6.3)	12.8 (4.6)	7.0 (1.2)	9.2 (3.2)	6.6 (7.6)
	CDA 1 (Amp Sum)	13.4 (6.1)	11.7 (4.8)	3.3 (2.3)	1.3 (4.5)	16.0 (6.4)
	CDA 2 (SCR)	15.2 (5.8)	9.5 (5.1)	3.0 (2.4)	1.1 (4.6)	12.1 (6.9)

*Peak score*	Magnitude	26.4 (4.3)	15.6 (4.1)	4.8 (2.0)	11.7 (2.6)	20.2 (5.9)

A peak score measure as recommended by the Society for Psychophysiological Research (Boucsein et al., 2012) was analysed for comparison (Fig. 1, Table 1). As previously shown (Bach et al., 2009, Bach et al., 2013), SA estimates from this approach had significantly lower sensitivity than the GLM estimates from SCRalyze. Ledalab measures showed no significant difference from peak scoring in most contrasts. In some contrasts, DDA measures were significantly less sensitive than peak scoring, and in two contrasts, CDA 2 was significantly more sensitive than peak scoring.

For analysis of within-subject contrasts, SCRalyze uses *z*-transformed data to eliminate between-subjects variance due to peripheral factors unrelated to the experimental conditions (e.g. skin properties) as an integral step in the analysis, while Ledalab does not do so. This might be a possible explanation for the higher sensitivity of SCRalyze, which I sought to explore in further analyses.

First, I *z*-transformed raw data before Ledalab analysis. Sensitivity of Ledalab measures did not improve consistently (see Table 1). However, SCRalyze *z*-transforms data after high-pass filtering, and this Ledalab analysis necessarily *z*-transformed before high-pass filtering. This means that variance in tonic skin conductance level does not influence *z*-transformation for SCRalyze because it is filtered out, but it does influence *z*-transformation for Ledalab. Hence, I performed a second analysis to only account for between-subject variance in phasic reactivity. To do so, I *z*-transformed the trial-by-trial estimates of phasic arousal before averaging within conditions (Table 1). This approach generally increased the predictive model evidence for the Ledalab estimates (i.e. reduced LBF); but the SA estimates from SCRalyze still performed significantly better in 4 out of 5 comparisons, and similar in the remaining one. These analyses suggest that *z*-transformation is one reason why SCRalyze performs better than Ledalab. Yet, even when removing this factor, SCRalyze is more sensitive than Ledalab such that other factors must be taken into account to explain this difference.

Specificity of the methods was also analysed. When comparing two random samples of trials from the same experimental condition, all methods yielded significant results at the rate prescribed by the error rate of the statistical test (1000 repetitions, expected number of significant tests: 50; observed number of significant tests for SCRalyze/DDA 1/DDA 2/CDA 1/CDA 2/Peak scoring: 53/60/55/47/56/57). Hence, all methods under study are unbiased with respect to differentiating two conditions without SA difference.

## 4 Discussion

In this paper, I compare two model-based methods for SCR analysis in their sensitivity to recover a known difference in SA between two conditions. In this head-to-head comparison, the GLM implemented in SCRalyze emerges to provide significantly higher predictive validity than any Ledalab measure in four out of five contrasts, and the same predictive validity as Ledalab's CDA measures in the fifth contrast. DDA measures were significantly less sensitive than GLM in all contrasts. At the same time, CDA and DDA did not perform consistently better than a standard peak scoring approach while GLM does.

Several reasons might account for the better sensitivity of SCRalyze. First, SCRalyze removes between-subject variance as a standard procedure, while Ledalab does not. Supplementary Ledalab analysis revealed that removing between-subjects variance improves performance of Ledalab, but SCRalyze is still significantly more sensitive. A second reason is the different modelling approach. Ledalab inverts the model SN → SCR and estimates an SN time series that (almost) perfectly explains the SCR time series. The method then proceeds to form operational SN indices of SA. SCRalyze, on the other hand, uses a probabilistic model SA → SN → SCR that seeks to only explain variance in the SCR data that is generated by the experiment, and discards the remaining data variance as noise. The software uses optimisation procedures to estimate the most likely SA, given observed data. A recent technical investigation of alternatives to the SCRalyze GLM has revealed that most models which explain more variance in the observed SCR data have worse sensitivity (Bach et al., 2013), despite their ability to better explain the data. A conclusion may be that those model-based methods which primarily focus on good data fit tend to attribute noise variance to underlying SA – which would then reduce the sensitivity of SA estimates. The GLM implemented in SCRalyze focuses on fitting only variance in the data that could have been caused by stimulus-locked SA – and discards the rest. Finally, I have previously shown that data conditioning can be optimised to improve SA estimation (Bach, Friston & Dolan, 2013). It remains to be shown whether optimised data conditioning can improve Ledalab to the point of being comparable to SCRalyze.

The higher sensitivity of SCRalyze to detect condition differences comes at no cost in terms of specificity. None of the methods under study was biased to detect condition differences in the absence of such differences.

Among SA indices derived with Ledalab, those based on DDA were consistently less sensitive than those based on CDA. DDA is recommended only for artefact-free data (Benedek and Kaernbach, 2010a, Benedek and Kaernbach, 2010b). Since no formal artefact rejection was performed in the present analysis, it might be possible that artefacts compromise DDA performance, and this could explain the better performance of CDA measures.

To summarise, a head-to-head comparison of SCRalyze (GLM) and Ledalab (DDA/CDA) reveals that SCRalyze provides better sensitivity than Ledalab measures for almost all comparisons of interest, and equal sensitivity for the remaining one, while at the same time providing better sensitivity than a peak-scoring approach. With this work, I hope to encourage independent research groups to compare methods for SCR analysis with respect to their sensitivity and thus foster further methodological developments.

## Conflict of interest

The author of this article is also the main developer of the software SCRalyze which was evaluated here.
